# Candidate germline polymorphisms of genes belonging to the pathways of four drugs used in osteosarcoma standard chemotherapy associated with risk, survival and toxicity in non-metastatic high-grade osteosarcoma

**DOI:** 10.18632/oncotarget.11486

**Published:** 2016-08-22

**Authors:** Claudia M. Hattinger, Paola Biason, Erika Iacoboni, Sara Gagno, Marilù Fanelli, Elisa Tavanti, Serena Vella, Stefano Ferrari, Andrea Roli, Rossana Roncato, Luciana Giodini, Katia Scotlandi, Piero Picci, Giuseppe Toffoli, Massimo Serra

**Affiliations:** ^1^ Laboratory of Experimental Oncology, Orthopaedic Rizzoli Institute, Bologna, Italy; ^2^ National Institute of Health and Medical Research (INSERM), Unity 892, University of Medicine of Angers, Angers, France; ^3^ Experimental and Clinical Pharmacology Unit, National Cancer Institute, Aviano, Italy; ^4^ Chemotherapy Ward of Muscoloskeletal Tumours, Orthopaedic Rizzoli Institute, Bologna, Italy; ^5^ Department of Computer Science and Engineering (DISI), University of Bologna, Cesena, Italy

**Keywords:** osteosarcoma, germline polymorphisms, toxicity, drug response biomarkers, personalized medicine

## Abstract

This study aimed to identify associations between germline polymorphisms and risk of high-grade osteosarcoma (HGOS) development, event-free survival (EFS) and toxicity in HGOS patients treated with neo-adjuvant chemotherapy and surgery.

Germline polymorphisms of 31 genes known to be relevant for transport or metabolism of all four drugs used in HGOS chemotherapy (methotrexate, doxorubicin, cisplatin and ifosfamide) were genotyped in 196 patients with HGOS and in 470 healthy age and gender-matched controls. Of these 196 HGOS patients, a homogeneously treated group of 126 patients was considered for survival analyses (survival cohort). For 57 of these, treatment-related toxicity data were available (toxicity cohort).

Eleven polymorphisms were associated with increased risk of developing HGOS (*p* < 0.05). The distribution of polymorphisms in patients was characterized by a higher Shannon entropy. In the survival cohort (*n* = 126, median follow-up = 126 months), genotypes of ABCC2_1249A/G, GGH_452T/C, TP53_IVS2+38G/C and CYP2B6*6 were associated with EFS (*p* < 0.05). In the toxicity cohort (*n* = 57), genotypes of ABCB1_1236T/C, ABCC2_1249A/G, ABCC2_3972A/G, ERCC1_8092T/G, XPD_23591A/G, XRCC3_18067T/C, MTHFR_1298A/C and GGH_16T/C were associated with elevated risk for toxicity development (*p* < 0.05).

The results obtained in this retrospective study indicate that the aforementioned germline polymorphisms significantly impact on the risk of HGOS development, EFS and the occurrence of chemotherapy-related toxicity. These findings should be prospectively validated with the aim of optimizing and tailoring HGOS treatment in the near future.

## INTRODUCTION

High-grade osteosarcoma (HGOS) is the most common malignant bone tumor arising in children and young adults. Current treatment of non-metastatic HGOS consists of surgical removal of the primary tumor and systemic pre- and post-operative multidrug chemotherapy. Standard protocols include doxorubicin, cisplatin, high-dose methotrexate and/or ifosfamide and/or etoposide, which allow to cure about 60–65% of patients with conventional HGOS (non-metatstatic at diagnosis, tumor localized in the extremities in patients younger than 40 years) [1–3].

Pharmacogenomic information regarding the drugs used in HGOS standard chemotherapy were obtained in other more frequent malignancies, and potentially common pathways and functional polymorphisms in these proteins that influence both chemotherapy toxicity and outcome were identified (*https://www.pharmgkb.org/*). On the other hand, a number of case-control studies revealed a series of polymorphisms associated with increased risk for HGOS development (*https://phgkb.cdc.gov/HuGENavigator/startPagePhenoPedia.do*, [4]) but none of these was confirmed by a recent international genome-wide-association-study approach [5].

The primary aim of this study was the identification of germline polymorphisms associated with HGOS development and comparison of their distribution in the cohort of controls (*n* = 470) with the one in the cohort of HGOS patients (*n* = 196) by means of information-theoretical measures. The secondary aim was the identification of polymorphisms with an impact on event-free survival (EFS) in a subgroup of 126 patients with conventional HGOS treated with neo-adjuvant chemotherapy, including methotrexate, doxorubicin, cisplatin and ifosfamide, and surgical removal of the primary tumor. The third aim consisted in the investigation of associations between genotypes and treatment-related toxicity in a subgroup of 57 patients with registered toxic events.

## RESULTS

To assess the effect of the different alleles of each polymorphism, data were classified as follows: “Wild-type” (WT) as homozygous *status* of the most frequent allele as reported in the reference public database (Table 1), “Hetero” (HET) as the heterozygous *status* of the variant allele and “Variant” (VAR) as the homozygous *status* of the variant allele.

**Table 1:** Characteristics of germline polymorphisms included in the study

Main gene function	Gene Name_Polymorphism	Reference SNP number	Type of change	Function	Effect on protein	Most frequent allele in database	Pubblic database used as reference
Transport	ABCB1_3435T/C	rs1045642	SNV	cds-synon	Ile1145Ile	T	HAPMAPCEU
	ABCB1_1236T/C	rs1128503	SNV	cds-synon	Gly412Gly	C	HAPMAPCEU
	ABCB1_2677G > T/A	rs2032582	SNV	missense	Ser893Thr	G	HAPMAPCEU
	ABCC2_1249A/G	rs2273697	SNV	missense	Val417Ile	G	HAPMAPCEU
	ABCC2_-24A/G	rs717620	SNV	UTR-5	NA	G	HAPMAPCEU
	ABCC2_3972A/G	rs3740066	SNV	cds-synon	Ile1092Ile	G	HAPMAPCEU
	ABCG2_34A/G	rs2231137	SNV	missense	Val12Met	G	HAPMAPCEU
	ABCG2_421A/C	rs2231142	SNV	missense	Gln141Lys	C	HAPMAPCEU
	RFC_80A/G	rs1051266	SNV	missense	His27Arg	G	HAPMAPCEU
DNA Repair	APE1_2197T/G	rs1130409	SNV	missense	Asp148Glu	G	HAPMAPCEU
	ERCC1_8092T/G	rs3212986	SNV	UTR-3	NA	G	HAPMAPCEU
	ERCC1_19007T/C	rs11615	SNV	cds-synon	Asn118Asn	T	HAPMAPCEU
	hMLH1_676A/G	rs1799977	SNV	missense	Ile219Ile	A	HAPMAPCEU
	hMSH2_IVS12-6T/C	rs2303428	SNV	splice region and intron variant	NA	T	HAPMAPCEU
	hOGG1_1245C/G	rs1052133	SNV	missense	Ser326Cys	C	HAPMAPCEU
	XPD_35931T/G	rs13181	SNV	missense	Lys727Gln	T	HAPMAPCEU
	XPD_23591A/G	rs1799793	SNV	missense	Asp288Asn	G	HAPMAPCEU
	XPG_3508G/C	rs17655^HW^	SNV	missense	Asp1104His	C	HAPMAPCEU
	XRCC1_28152A/G	rs25487	SNV	missense	Gln399Arg	G	HAPMAPCEU
	XRCC3_18067T/C	rs861539	SNV	missense	Thr241Met	C	HAPMAPCEU
Folate cycle	DHFR_Ins/Del	rs70991108	DIV	nearGene-5/1st intron	NA	Ins	[6]
	FOLR1_181delC	rs3833748	DIV	nearGene-5	NA	I	CAUC1
	GGH_452T/C	rs11545078	SNV	missense	Thr151Ile	C	HAPMAPCEU
	GGH_401T/C	rs3758149^HW^	SNV	nearGene-5	NA	C	HAPMAPCEU
	GGH_16T/C	rs1800909^HW^	SNV	missense	Cys6Arg	T	PILOT1
	MTHFD1_1958T/C	rs2236225	SNV	missense	Arg653Gln	C	HAPMAPCEU
	MTHFR_677T/C	rs1801133	SNV	missense	Ala222Val	C	HAPMAPCEU
	MTHFR_1298A/C	rs1801131	SNV	missense	Glu429Ala	A	HAPMAPCEU
	SHMT_1420T/C	rs2273029	SNV	intron variant	NA	C	HAPMAPCEU
	TYMS_28bp_VNTR	rs34743033	STR	NA	NA	3 (repeats)	CAUC1
	TYMS_1494del6	rs16430	DIV	6 bp deletion in micro-RNA binding site	NA	NA	excluded from SNPdb
Apoptosis	ATM_40C/G	rs1800054	SNV	missense	Ser49Cys	C	CAUC1
	ATM_61A/G	rs1801516	SNV	missense	Asp1853Asn	G	HAPMAPCEU
	MDM2_309T/G	rs2279744	SNV	intron variant	NA	T	PILOT1
	p21_98A/C	rs1801270	SNV	missense	Ser31Arg	C	HAPMAPCEU
	TP53_PIN3_IVS3 + 16 bp	rs17878362	STR	NA	NA	NA	no frequence data available
	TP53_IVS2+38G/C	rs1642785	SNV	UTR-5	NA	G	PILOT1
	TP53_ex4+119G/C	rs1042522^HW^	SNV	missense	Pro72Arg	G	HAPMAPCEU
Drug metabolism	GSTT1_gen_null	rs_GSTT1_gen_null	DIV	reduced or no protein activity	NA	null	[7]
	GSTM1_gen_null	rs_GSTM1_gen_null	DIV	reduced or no protein activity	NA	non-null	[7]
	GSTP1_313A/G	rs1695	SNV	missense	Ile105Val	A	HAPMAPCEU
	CYP2C19*2_681A/G	rs4244285	SNV	cds-synon	Pro227Pro	G	HAPMAPCEU
	CYP2B6*6(516T/G + 785A/G)	rs3745274 and rs2279343	Haplotype	reduced protein expression	Gln172His; Lys262Arg	NA	[8]
	CYP2B6*7(*6 + 1459T/C)	rs3745274 and rs2279343 and rs3211371	Haplotype	reduced protein expression	Gln172His; Lys262Arg; Arg487Cys	NA	[8]
	CYP2C9*2_430T/C	rs1799853	SNV	missense	Arg144Cys	C	HAPMAPCEU
	CYP2C9*3_1075A/C	rs1057910	SNV	missense	Ile359Leu	A	HAPMAPCEU
	CYP3A4*1B_-392A/G	rs2740574	SNV	nearGene-5	NA	A	EGP_CEPH-PANEL

HW: polymorphism with significant deviation from Hardy-Weinberg equilibrium *(P* < 0.01); SNV: single nucleotide variation, DIV: deletion/insertion variation, STR: short tandem repeat (microsatellite) variation, cds-synon: coding region - synonymous, UTR: untranslated region, NA: not applicable.

In order to enable 2×2 table and logistic regression analyses genotypic data were also categorized in two groups according to 1) the dominant model: WT *versus* HET + VAR and 2) the recessive model: WT + HET *versus* VAR. For three polymorphisms without public frequency data, WT was classified as follows: DHFR_Ins/Del (rs70991108) and TYMS_1494del6 (rs16430) homozygous without deletion; TP53_PIN_IVS3+16bp (rs17878362) homozygous without insertion.

### Genotypes associated with risk for osteosarcoma development

Frequencies of the WT, HET and VAR *status* of all polymorphisms were calculated for the healthy controls (*n* = 470) and the total cohort of patients with HGOS (*n* = 196) (Table 2). Genotype frequencies of controls were similar to those reported in public databases demonstrating its representativeness.

**Table 2:** Genotype frequencies in 196 osteosarcoma patients and 470 healthy controls

Main Gene Function	Polymorphism	196 Patients			470 Healthy controls			
		WT (%)	HET (%)	VAR (%)	WT (%)	HET (%)	VAR (%)	*p*
TRANSPORT	ABCB1_3435T/C	26	44	30	24	52	24	
	ABCB1_1236T/C	32	45	23	33	50	17	
	ABCB1_2677G/T/A	29	48	23	28	53	19	
	**ABCC2_1249A/G**	**50**	**46**	**4**	**63**	**34**	**3**	**0.011**
	ABCC2_-24A/G	60	37	3	66	30	4	
	**ABCC2_3972A/G**	**46**	**36**	**18**	**47**	**44**	**9**	**0.030**
	ABCG2_34A/G	84	16	0	88	11	1	
	ABCG2_421A/C	85	13	2	80	19	1	
	RFC_80A/G	39	45	16	37	49	14	
DNA REPAIR	APE1_2197T/G	16	50	34	20	49	31	
	ERCC1_8092T/G	62	32	6	54	39	7	
	ERCC1_19007T/C	34	43	23	35	47	18	
	**hMLH1_676A/G**	**33**	**54**	**13**	**45**	**41**	**14**	**0.010**
	hMSH2_IVS12-6T/C	85	14	1	86	13	1	
	hOGG1_1245C/G	65	29	6	63	33	4	
	XPD_35931T/G	40	45	15	38	46	16	
	XPD_23591A/G	43	40	17	44	41	15	
	**XPG_3508G/C**	**57**	**31**	**12**	**54**	**41**	**5**	**0.002**
	XRCC1_28152A/G	42	44	14	44	45	11	
	XRCC3_18067T/C	32	51	17	38	44	18	
FOLATE CYCLE	DHFR_Ins/Del	34	50	16	35	55	10	
	FOLR1_181delC	96	4	0	97	3	0	
	GGH_452T/C	82	15	3	79	20	1	
	**GGH_401T/C**	**53**	**31**	**16**	**52**	**40**	**8**	**0.042**
	**GGH_16T/C**	**63**	**22**	**15**	**57**	**35**	**8**	**0.011**
	MTHFD1_1958T/C	27	53	20	28	50	22	
	MTHFR_677T/C	30	49	21	32	55	13	
	MTHFR_1298A/C	47	44	9	41	47	12	
	SHMT_1420T/C	52	38	10	58	34	8	
	**TYMS_28bp_VNTR**	**44**	**39**	**17**	**32**	**45**	**23**	**0.012**
	TYMS_1494del6	30	54	16	37	47	16	
APOPTOSIS	ATM_40C/G	98	2	0	96	4	0	
	ATM_61A/G	72	25	3	77	22	1	
	**MDM2_309T/G**	**34**	**42**	**24**	**47**	**41**	**12**	**< 0.001**
	p21_98A/C	84	15	1	85	14	1	
	TP53_PIN3_IVS3+16bp	68	25	7	69	27	4	
	**TP53_IVS2+38G/C**	**8**	**33**	**59**	**56**	**36**	**8**	**< 0.0001**
	**TP53_ex4+119G/C**	**71**	**21**	**8**	**59**	**34**	**7**	**0.007**
DRUG METABOLISM	GSTT1_gen_null	84	0	16	81	0	19	
	GSTM1_gen_null	43	0	57	47	0	53	
	GSTP1_313A/G	48	44	8	51	43	6	
	CYP2C19*2_681A/G	74	23	3	73	25	2	
	CYP2B6*6	**66**	**27**	**7**	**58**	**38**	**4**	**0.030**
	CYP2B6*7	**99**	**1**	**0**	**96**	**4**	**0**	**0.032**
	CYP2C9*2_430T/C	75	23	2	72	26	2	
	CYP2C9*3_1075A/C	86	12	2	82	17	1	
	CYP3A4*1B_-392A/G	91	9	0	95	5	0	

Those polymorphisms that resulted to be significantly distributed differently in osteosarcoma patients compared to healthy controls are given in bold with their two-sided *p* values. WT, wild-type; HET, heterozygous; VAR, variant.

Contingency analyses identified 12 polymorphisms that were significantly differently distributed in HGOS patients relative to healthy controls (Table 2). In order to obtain larger group sizes and thus more robust results, patients were grouped according to either the dominant or recessive genotypic model. These analyses identified one additional polymorphism MTHFR _677T/C (VAR versus WT + HET, *p* = 0.021) that was significantly differently distributed in HGOS patients compared to healthy controls.

**Figure 1:**
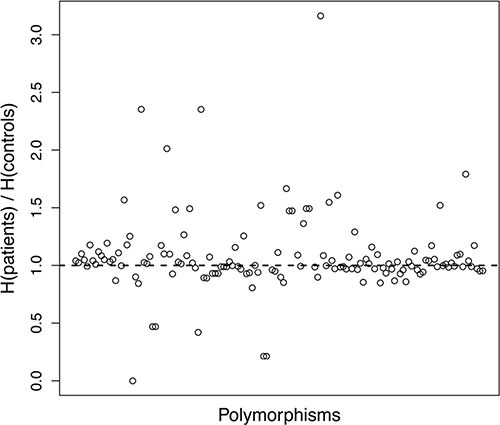
Graphical representation of Shannon entropy For each polymorphism and each classification type, the ratio of patient and control entropies is plotted. Each circle in the plot represents the ratio between the entropy values of patients and controls for a specific polymorphism. For the sake of readability, the identity of polymorphisms is not reported, as it is not relevant for the purpose of this analysis.

Shannon entropy was computed for all the polymorphisms and all the three genotypic models (Figure 1). The difference between the two cohorts was remarkable: the entropy in patients was higher than the one in controls in 52% of the cases, while it was lower in 45% of the cases. In the remaining 3% the entropy values were equal. Besides the simple counting of cases with higher/lower entropy, it is important to observe that the cases with higher patient entropy seem to be characterized by a larger difference of entropy values. To assess this hypothesis, we applied a Wilcoxon test with a 95% confidence level. The resulting *p* value of 0.021 provided significant evidence that the entropy of the polymorphism distribution in patients was higher than in controls. The most notable differences with *r*, which is the ratio between patients and controls entropy of a given polymorphism distribution, greater than 1.5 were found for the following polymorphisms in the recessive model: ABCC2_3972A/G, ABCG2_421A/C, ATM_61A/G, TP53_IVS2+38G/C, CYP2B6*6, GGH_452T/C, TP53_IVS2+38G/C, CYP2C9*3_1075A/C, GGH_452T/C, GGH_401T/C, GGH_16T/C, hOGG1_1245C/G and XPG_3508G/C.

Cluster index analysis was applied and revealed that the average values of Tc (which is a clustering significance measure and was computed as described in detail in the supplementary information) were much higher in controls then in patients. In general, the highest Tc values in controls were about three times the Tc values of patients. This outcome provided further evidence supporting the fact that the distribution of polymorphisms in patients was more heterogeneous than in controls.

To determine whether the differently distributed polymorphisms were associated also with an increased risk for HGOS development, simple logistic regression analysis was performed on the data according to the dominant or recessive genotypic model. Eleven polymorphisms resulted to be associated with increased risk for HGOS development (Table 3).

**Table 3:** Genotypes significantly associated with increased relative risk to develop osteosarcoma compared to healthy controls identified by logistic regression analysis

Polymorphism [risk-associated genotype]	OR (CI 95%)	*p*
ABCC2_1249A/G [HET + VAR]	1.71 (1.20–2.43)	0.003
ABCC2_3972A/G [VAR]	2.24 (1.18–4.24)	0.013
hMLH1_676A/G [HET + VAR]	1.62 (1.14–2.31)	0.007
XPG_3508G/C [VAR]	2.47 (1.36–4.50)	0.003
GGH_401T/C [VAR]	2.14 (1.07–4.69)	0.032
GGH_16T/C [VAR]	2.24 (1.07–4.69)	0.033
MTHFR_677T/C [VAR]	1.68 (1.08–2.61)	0.022
TYMS_28bp_VNTR [HET + VAR]	1.67 (1.18–2.35)	0.004
MDM2_309T/G [HET + VAR]	1.73 (1.22–2.46)	0.002
[VAR]	2.25 (1.46–3.47)	< 0.001
TP53_IVS2 + 38G/C [HET + VAR]	13.37 (7.76–23.03)	< 0.0001
[VAR]	15.63 (10.20–24.39)	< 0.0001
TP53_ex4 + 119G/C [WT]	1.68 (1.17–2.41)	0.005

OR, odd ratio; CI, confidence interval; *p* values refer to the genotype with higher risk which is given in brackets; WT, wild-type; HET, heterozygous; VAR, variant.

### Genotypes with impact on event-free survival in osteosarcoma patients

To identify genotypes with impact on clinical outcome, survival analyses were performed for each polymorphism in a subgroup of 126 HGOS patients with homogeneous clinico-pathological features and treatment (Table 4). This subgroup was representative of the whole patient series since frequency distributions of all genotypes and clinico-pathological parameters were similar to those of all the 196 patients (Tables 2 and 5).

**Table 4:** Clinical and pathological characteristics of study patients and cohorts included in survival and toxicity analyses

Characteristics	Total cohort (frequency) *n* = 196	Survival cohort (frequency) *n* = 126	Toxicity cohort (frequency) *n* = 57
**Gender**			
Male	122 (62%)	75 (60%)	37 (65%)
Female	74 (38%)	51 (40%)	20 (35%)
**Age**			
< 14 years	60 (31%)	43 (34%)	24 (42%)
≥ 14 years	136 (69%)	83 (66%)	33 (58%)
**Site**			
Femur	101 (51%)	75 (60%)	30 (53%)
Tibia	39 (20%)	27 (21%)	15 (26%)
Humerus	23 (12%)	15 (12%)	9 (16%)
Fibula	8 (4%)	5 (4%)	3 (5%)
Other	25 (13%)	4 (3%)	0 (0%)
**Metastasis at diagnosis**			
Absent	179 (91%)	126 (100%)	57 (100%)
Present	17 (9%)	0 (0%)	0 (0%)
**Histologic Subtype**			
Osteoblastic	125 (64%)	88 (70%)	43 (75%)
Fibroblastic	24 (12%)	19 (15%)	6 (11%)
Chondroblastic	18 (9%)	14 (11%)	7 (12%)
Telangiectatic	3 (2%)	2 (2%)	1 (2%)
Not specified	22 (11%)	3 (2%)	0 (0%)
Irradiation-induced	4 (2%)	0 (0%)	0 (0%)
**Surgery**			
Yes	194 (99%)	126 (100%)	57 (100%)
No	2 (1%)	0 (0%)	0 (0%)
**Treatment**			
Adjuvant^a^	16 (8%)	0 (0%)	0 (0%)
Neo-adjuvant^b^	171 (87%)	126 (100%)	57 (100%)
No chemotherapy	9 (5%)	0 (0%)	0 (0%)

a Treatment including doxorubicin, cisplatin and methotrexate.

b Treatment including doxorubicin, cisplatin, methotrexate and ifosfamide.

**Table 5:** Genotype frequencies in the subgroups of osteosarcoma patients eligible for survival (survival cohort) and toxicity (toxicity cohort) analyses

Main gene function	Polymorphism	Survival cohort (*n* = 126 patients)			Toxicity cohort (*n* = 57 patients)		
		WT (%)	HET (%)	VAR (%)	WT (%)	HET (%)	VAR (%)
TRANSPORT	ABCB1_3435T/C	29	44	27	33	44	23
	ABCB1_1236T/C	31	46	23	29	51	20
	ABCB1_2677G/T/A	27	46	27	21	49	30
	ABCC2_1249A/G	49	48	3	54	44	2
	ABCC2_-24A/G	59	37	4	57	39	4
	ABCC2_3972A/G	48	35	17	37	41	22
	ABCG2_34A/G	85	15	0	91	9	0
	ABCG2_421A/C	82	16	2	85	15	0
	RFC_80A/G	37	46	17	37	51	12
DNA REPAIR	APE1_2197T/G	18	47	35	16	52	32
	ERCC1_8092T/G	60	34	6	60	38	2
	ERCC1_19007T/C	30	46	24	26	56	18
	hMLH1_676A/G	37	51	12	36	51	13
	hMSH2_IVS12-6T/C	89	11	0	89	11	0
	hOGG1_1245C/G	64	28	8	68	25	7
	XPD_35931T/G	38	49	13	37	52	11
	XPD_23591A/G	40	47	13	39	52	9
	XPG_3508G/C	57	31	12	65	30	5
	XRCC1_28152A/G	42	46	12	35	49	16
	XRCC3_18067T/C	30	55	15	27	58	15
FOLATE CYCLE	DHFR_Ins/Del	38	46	16	30	52	18
	FOLR1_181delC	94	6	0	98	2	0
	GGH_452T/C	82	15	3	83	12	5
	GGH_401T/C	49	35	16	55	29	16
	GGH_16T/C	61	24	15	66	19	15
	MTHFD1_1958T/C	28	55	17	27	57	16
	MTHFR_677T/C	31	52	17	33	51	16
	MTHFR_1298A/C	41	49	10	39	50	11
	SHMT_1420T/C	53	40	7	50	41	9
	TYMS_28bp_VNTR	39	43	18	30	47	23
	TYMS_1494del6	33	53	14	30	54	16
APOPTOSIS	ATM_40C/G	98	2	0	98	2	0
	ATM_61A/G	72	26	2	73	25	2
	MDM2_309T/G	30	50	20	28	47	25
	p21_98A/C	87	13	0	93	7	0
	TP53_PIN3_IVS3 + 16 bp	66	29	5	63	35	2
	TP53_IVS2+38G/C	7	35	58	9	34	57
	TP53_ex4 + 119G/C	66	27	7	70	21	9
DRUG METABOLISM	GSTT1_gen_null	83	0	17	88	0	12
	GSTM1_gen_null	42	0	58	39	0	61
	GSTP1_313A/G	52	42	6	54	44	2
	CYP2C19*2_681A/G	75	24	1	72	26	2
	CYP2B6*6	65	29	6	68	25	7
	CYP2B6*7	99	1	0	100	0	0
	CYP2C9*2_430T/C	73	26	1	68	32	0
	CYP2C9*3_1075A/C	86	11	3	91	9	0
	CYP3A4*1B_-392A/G	90	10	0	89	11	0

WT, wild-type; HET, heterozygous; VAR, variant.

**Figure 2:**
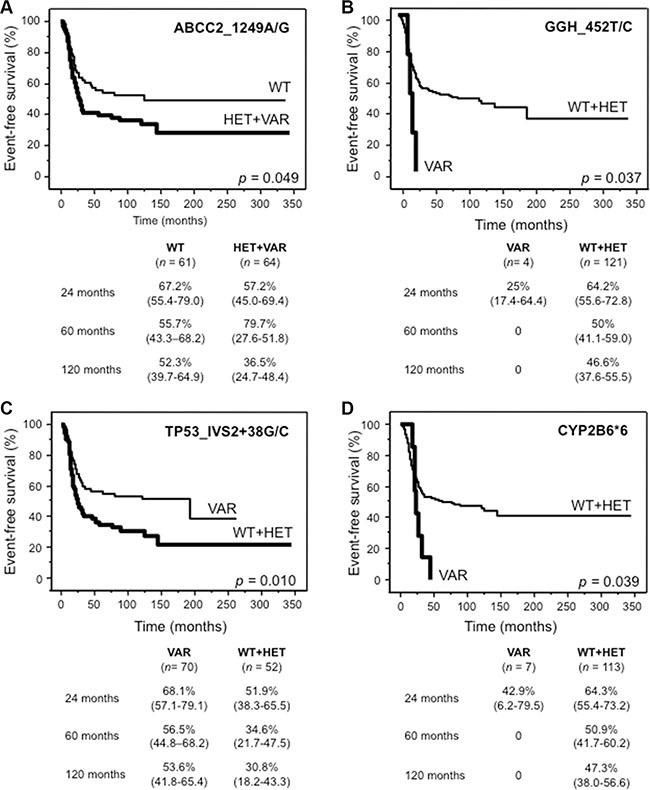
Kaplan Meier curves for the four genotypes significantly influencing event-free survival in univariate analysis (**A**) ABCC2_1249A/G (rs2273697), (**B**) GGH_452T/C (rs11545078), (**C**) TP53_IVS2+38G/C (rs1642785) and (**D**) CYP2B6*6. For each genotype, tables show survival probabilities in % with 95% confidence interval in parenthesis at 24, 60 and 120 months for both categories. *n*, number of patients; WT, wild-type; HET + VAR, heterozygous + variant; WT + HET, wild-type + heterozygous; VAR, variant.

Four polymorphisms, ABCC2_1249A/G, GGH_452T/C, TP53IVS2 + 38C > G and CYP2B6*6, were significantly associated with EFS in univariate analysis (Figure 2). Worse EFS was associated with the genotypes HET + VAR of ABCC2_1249A/G (A), VAR of GGH_452T/C (B), WT + HET of TP53IVS2 + 38C > G (C) and VAR of CYP2B6*6 (D). However none of these genotypes, which we defined as “risk genotypes”, retained statistical significance by multiparametric Cox proportional hazards regression analysis (data not shown). By using the true median EFS times as input data, all four polymorphisms reached a power of at least 55% (range 46–100%) (type I error 5%) to detect survival differences between the two groups characterized by the different genotypes.

**Figure 3:**
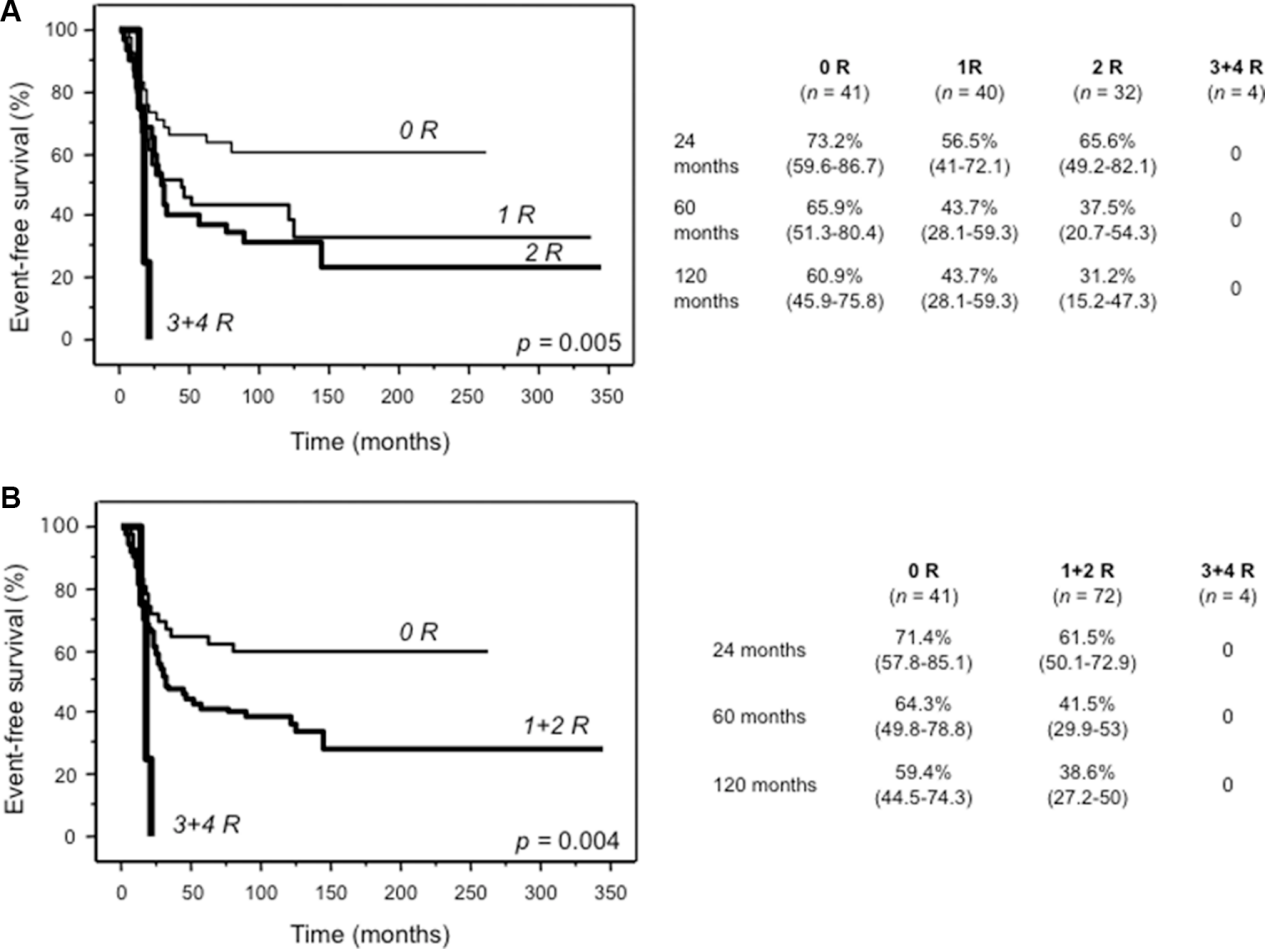
Kaplan Meier curves of event-free survival for 117 patients assigned to four (A) or three (B) risk groups defined by the presence of none, 1, 2, 3 or 4 risk genotypes of ABCC2_1249A/G (rs2273697), GGH_452T/C (rs11545078), TP53_IVS2+38G/C (rs1642785) and CYP2B6*6 For each risk group (0 R, 1 R, 2 R, 3 + 4 R), tables show survival probabilities in % with 95% confidence interval in parenthesis at 24, 60 and 120 months for all categories. *n*, number of patients.

Based on the evidence that the aforementioned risk genotypes individually impacted on EFS, we stratified patients into four risk groups. Nine patients were excluded because they did not have data of all these four polymorphisms. As shown in Figure 3, patients carrying three or four risk genotypes (*n* = 4) performed significantly worse, compared to patients with two (*n* = 32), one (*n* = 40) or none (*n* = 41) risk genotypes (A). This difference was even more evident when patients with one or two risk genotypes were grouped together (B).

None of the genotypes resulted in a statistically significant association with any of the clinical parameters shown in Table 4, which did also not impact on survival (data not shown).

### Toxicity associated with genotypes in osteosarcoma patients

Possible associations between chemotherapy-related toxicity and polymorphisms were evaluated in a subgroup of 57 patients enroled in the ISG/OS-1 protocol. For these analyses, genotype data were categorized according to the dominant model in WT and HET + VAR, in order to avoid too small sample sizes.

Eight polymorphisms proved to be significantly associated with toxic events (Table 6). Significant predictivity was confirmed for five of them by multiple logistic regression analysis (Table 6). Neurotoxicity was registered in four patients (7%), but no predictive polymorphism was identified. Nephrotoxicity and stomatitis were registered in one patient each (2%) and were therefore not included in association analyses.

**Table 6:** Toxic events associated with genotypes in 57 osteosarcoma patients and relative risk estimation by logistic regression analyses

Toxic events	Frequency of toxic events	Polymorphism	*p* (Fisher's Test)	Univariate logistic regression		Multivariate logistic regression	
				OR (95% CI)	*p*	Adjusted OR (95% CI)	*p*
Leukopenia grade 4	79%	ABCC2_1249A/G [HET + VAR] MTHFR_1298A/C [HET + VAR]	0.007 0.047	13.16 (1.56–111.12) 4.15 (1.06–16.13)	0.018 0.040	10.20 (1.17–90.90) 2.93 (0.69–12.50)	0.035 0.145
Thrombocytopenia grade 4	68%	ABCC2_1249A/G [HET + VAR] XPD_23591A/G [HET + VAR]	0.024 0.004	4.33 (1.20–15.63) 5.59 (1.66–18.87)	0.025 0.006	23.81 (2.50–250.00) 27.78 (3.10–250.00)	0.006 0.003
Red blood cells transfusion	54%	ABCC2_1249A/G [HET + VAR]	0.002	6.06 (1.88–19.60)	0.003		
Platelet transfusion	39%	ABCC2_1249A/G [HET + VAR]	0.030	3.36 (1.10–10.20)	0.033		
Fever	70%	ERCC1_8092T/G [HET + VAR]	0.016	6.49 (1.30–32.26)	0.023		
Nausea/Vomiting	44%	ABCC2_3972A/G [WT] XRCC3_18067T/C [HET + VAR]	0.001 0.037	8.33 (2.35–29.58) 4.42 (1.08–18.18)	0.001 0.039	5.85 (1.57–21.85) 3.22 (0.56–18.52)	0.009 0.188
Hepatotoxicity (Transaminases grade 4)	56%	ABCB1_1236T/C [HET + VAR] ABCC2_1249A/G [WT] GGH_16T/C [WT]	0.047 0.050 0.025	2.06 (1.16–3.66) 1.96 (1.20–3.20) 2.87 (1.53–5.41)	0.014 0.007 0.001	2.28 (1.23–4.26) 1.83 (1.10–3.05) 2.95 (1.56–5.59)	0.009 0.021 0.001

OR, odd ratio; CI, confidence intervals; WT, wild-type; HET + VAR, heterozygous + variant; *p* values refer to the genotype with higher risk which is given in brackets.

Because of the different toxicity profile of methotrexate compared to doxorubicin, cisplatin and ifosfamide, logistic regression analyses were performed to estimate the relative risk for the occurrence of toxic events in relation to the drugs administered (Table 7). Considering methotrexate cycles, WT *status* of ABCC2_3972A/G was associated with nausea and vomiting, as well as ABCB1_1236T/C (HET + VAR), ABCC2_1249 (WT) and GGH_16T/C (WT) with transaminases increase grade 4. All three polymorphisms remained significant for transaminases increase grade 4 by multiple regression analysis (Table 7).

**Table 7:** Toxic events significantly associated with genotypes in relation to methotrexate or doxorubicin, cisplatin and ifosfamide by logistic regression analyses

Drug (cycles)	Toxic events	Polymorphism	Univariate logistic regression		Multivariate logistic regression	
			OR (95% CI)	*p*	Adjusted OR (95% CI)	*p*
Methotrexate (*n* = 520)	Nausea/Vomiting	ABCC2_3972A/G [WT]	3.15 (1.06–9.37)	0.039		
	Hepatotoxicity (Transaminases grade 4)	ABCB1_1236T/C [HET + VAR] ABCC2_1249A/G [WT] GGH_16T/C [WT]	2.21 (1.22–4.00) 2.48 (1.47–4.18) 2.93 (1.53–5.62)	0.009 0.001 0.001	2.46 (1.29–4.72) 2.28 (1.31–3.96) 3.05 (1.57–5. 92)	0.001 0.004 0.001
Doxorubicin/ Cisplatin/ Ifosfamide (*n* = 488)	Leukopenia grade 4	ABCC2_1249A/G [HET + VAR] MTHFR_1298A/C [HET + VAR]	2.80 (1.63–4.83) 2.19 (1.26–3.82)	< 0.001 0.006	2.19 (1.23–3.92) 1.75 (0.97–3.13)	0.008 0.063
	Thrombocytopenia grade 4 Red blood cells transfusion Platelet transfusion Nausea/Vomiting	ABCC2_1249A/G [HET + VAR] ABCC2_1249A/G [HET + VAR] ABCC2_1249A/G [HET + VAR] ABCC2_3972A/G [WT]	4.35 (2.17–8.77) 2.13 (1.12–4.07) 3.55 (1.59–7.87) 2.28 (1.02–5.07)	< 0.0001 0.022 0.002 0.045		

OR, Odd ratio; CI: confidence interval; *p* values refer to the genotype with higher risk which is given in brackets; WT, wild-type; HET + VAR, heterozygous + variant.

Regarding doxorubicin, cisplatin and ifosfamide cycles, a higher risk for leukopenia was associated with the presence of at least one variant allele of ABCC2_1249A/G and MTHFR_1298A/C. ABCC2_1249A/G (HET + VAR) was associated also with thrombocytopenia and the necessity of red blood cells or platelet transfusions (Table 7). Likewise for methotrexate cycles, a higher risk for nausea and vomiting was found for patients with WT *status* of ABCC2_3972A/G.

Multiple regression analysis confirmed the predictive value of the ABCC2_1249A/G genotype for leukopenia (Table 7).

## DISCUSSION

Although high-grade HGOS is the most common malignant bone tumor in children and adolescents, pharmacogenomic markers still cannot be used for patient stratification or treatment modulation [9]. Since scarce response to multi-drug chemotherapy occurs in about 40% of HGOS patients, and the four backbone drugs of HGOS chemotherapy can cause severe short- and long-time toxic events [10], there is urgent need to identify pharmacogenetic markers that can identify patients with higher risk for unresponsiveness and/or to develop drug-related toxicity.

More than 100 polymorphisms were reported in association with HGOS [4], but few of them were correlated with clinical parameters, survival and the development of toxic events, e.g. [9, 11, 12]. Therefore, we aimed to analyze polymorphisms known to be involved in the pathways of all four drugs used in HGOS chemotherapy in a well documented series of HGOS patients in order to address all these questions at the same time.

We observed 12 polymorphisms associated with an increased risk for HGOS development including five polymorphisms of genes being important in the folate metabolism, two polymorphisms of DNA repair genes, two polymorphisms of the ABCC2 transporter, and three of the apoptosis pathway. The variant allele of the MDM2_309T/G polymorphism was reported to be associated with sarcomas, including osteosarcoma, by several studies including meta-analyses [13–15]. The wild-type allele of TP53_ex4+119G/C was reported in association with risk of HGOS in two studies [16, 17] but not confirmed in one meta-analysis [13].

With respect to survival-related genotypes, the HET + VAR *status* of ABCC2_1249A/G, the VAR *status* of GGH_452T/C and CYP2B6*6 and the WT + HET *status* of TP53_IVS2+38G/C were associated with worse EFS. Given that these genes do not belong to the same pathway and are not reciprocally associated, a co-evaluation identified three groups with significantly different survival probabilities suggesting that these four polymorphisms have a combined effect on survival. To our knowledge, these polymorphisms have not been reported as prognostic factors in HGOS patients so far, but a similar approach has recently identified five other polymorphisms with combined effect on EFS [18]. On the other hand, several studies reported polymorphisms associated with survival that were analyzed also in our study, although they did not reveal any prognostic impact (e.g. ABCB1_1236T/C [11], drug-metabolizing enzymes [19]). The observation that different polymorphisms can impact similarly on EFS could be explained by the genomic complexity and heterogeneity of HGOS, by the different drugs used for treatment, nonetheless by the small number of overlapping polymorphisms analyzed in the different studies.

Doxorubicin, methotrexate and cisplatin are known substrates of ABCC2 and this protein was the third most expressed ABC transporter in a series of HGOS cell lines [20], although ABCB1 is the best described biological prognostic marker in HGOS [3]. Polymorphisms of *ABCC2* are known to influence the bioavailability of drugs and therefore could contribute to a reduced chemotherapy efficacy [21, 22].

GGH catalyzes the removal of polyglutamates, preferencially from long-chain methotrexate polyglutamates, thus influencing the overall effectiveness of methotrexate [23]. The VAR *status* of GGH_452T/C was found in children with acute lymphoblastic leukemia who showed exclusively low or intermediate catalytic activity of GGH with the consequence of intracellular accumulation of long-chain methotrexate polyglutamates [24].

Our data suggest that in HGOS this polymorphism plays a different role, since they indicated that the VAR *status* of GGH_452T/C might reduce the effectiveness of methotrexate treatment in HGOS patients. It would be therefore interesting to analyze whether the GGH_452T/C variant affects the hydrolytic activity of this enzyme also in HGOS cells.

Since the VAR *status* of CYP2B6*6 was described to result in decreased expression and activity of this enzyme in liver cells [8, 25], patients with HGOS might be less responsive to ifosfamide, which is activated by CYP2B6.

Regarding the role of TP53_IVS2+38G/C, we hypothesize that although the VAR *status* is associated with a high risk for HGOS development, patients seem to respond better to treatment and therefore have a higher probability of EFS compared to those with WT + HET *status*. However, these four polymorphisms were not associated with necrosis evaluated after pre-operative chemotherapy (data not shown) and differed from those described in other studies ([12, 18], drug-metabolizing enzymes, for review see [19]). Therefore the direct impact on survival needs still to be elucidated.

Among the polymorphisms associated with hematological toxicity, the HET + VAR *status* of ABCC2_1249A/G was associated with leukopenia and thrombocytopenia grade 4 and the necessity of red blood cells and platelet transfusion. This association remained significant even in multiple regression analysis and was associated prevalently to doxorubicin/cisplatin/ifosfamide treatment which can be ascribed to the fact that both doxorubicin and cisplatin are substrates of ABCC2.

Another variant associated with leukopenia grade 4 was the HET + VAR *status* of MTHFR_1298A/C, which was reported in association with anemia after methotrexate cycles in HGOS patients [12].

Hepatotoxicity was associated with the WT *status* of ABCC2_1249A/G and GGH_16T/C and with the HET + VAR *status* of ABCB1_1236T/C. All three polymorphisms remained significant by multiple regression analyses in relation to the complete treatment and to the methotrexate administration. Since polymorphisms of ABC-transporters are known to influence the bioavailability of several drugs [26], and methotrexate is substrate of ABCB1 and ABCC2, our findings suggest that the variant allele of ABCB1_1236T/C increases toxicity in hepatic cells due to a lower activity of the enzyme. Conversely, the variant allele of ABCC2_1249A/G is protective. Since there are no pharmacokinetic studies available in HGOS patients these assumptions need confirmation by functional studies.

To our knowledge, the functional role of GGH_16T/C polymorphism is still unknown. Our observation suggests that WT status could be associated with higher accumulation of methotrexate long chain polyglutamates, thus increasing cytotoxicity in hepatic cells.

Independently of the administered drugs, WT *status* of ABCC2_3972A/G was associated with nausea and vomiting. The mechanism underlying this association has yet to be elucidated since no data were reported.

A peculiar feature of this study was the application of the information-theoretical measures Shannon entropy and cluster analysis to genotyping data to support the interpretation of polymorphism distributions in patients and healthy controls by objective means. It is noteworthy, that among the eleven polymorphisms with *r* > 1.5, four were associated with risk to develop HGOS, three with impact on survival, and two with toxicity.

In order to obtain information regarding the potential functional effects of the polymorphisms analyzed in the present study, two web-based SNP bioinformatic tools were explored to predict their regulatory potential, as indicated by specific *in silico* scores (<https://snpinfo.niehs.nih.gov/snpinfo/snpfunc.htm>; <http://compbio.cs.queensu.ca/F-SNP/> and references therein). Several of the polymorphisms that resulted to be associated with survival, toxicity and/or risk for HGOS in this study also showed scores > 0.5 that indicated a relevant functional impact. This group included three polymorphisms associated with survival (ABCC2_1249A/G, GGH_452T/C, CYP2B6*6), seven associated with toxicity (ABCC2_1249A/G, ABCC2_3972A/G, ERCC1_8092T/G, XPD_23591A/G, XRCC3_18067T/C, GGH_16T/C, MTHFR_1298A/C), and three associated with risk for HGOS (ABCC2_1249A/G, ABCC2_3972A/G, GGH_16T/C). This evidence indicates that the polymorphisms emerged in our study as associated to survival, toxicity and/or risk for HGOS also have a predicted relevant functional impact, which however needs to be explored and confirmed in further functional studies.

In summary, since the ABCC2_1249A/G polymorphism was associated with risk of HGOS development, worse EFS and hematological toxicity, and presented a relevant predicted functional impact, it should be prioritized for further validation studies in order to definitely estimate its relevance for a possible use as patients stratification marker. Furthermore, our data indicate to consider in addition to ABCC2_1249A/G, also TP53_IVS2+38G/C, GGH_452T/C and CYP2B6*6 in a prospective screening because of their demonstrated prognostic value. ABCB1_1236T/C, ABCC2_3972A/G, XPD_23591A/G, ERCC1_8092T/G, XRCC3_18067T/C, MTHFR_1298A/C, GGH_16T/C polymorphisms should be further validated within a prospective trial with registered toxic events to fully determine their value as markers for patient stratification.

## MATERIALS AND METHODS

### Patients and study design

**Figure 4:**
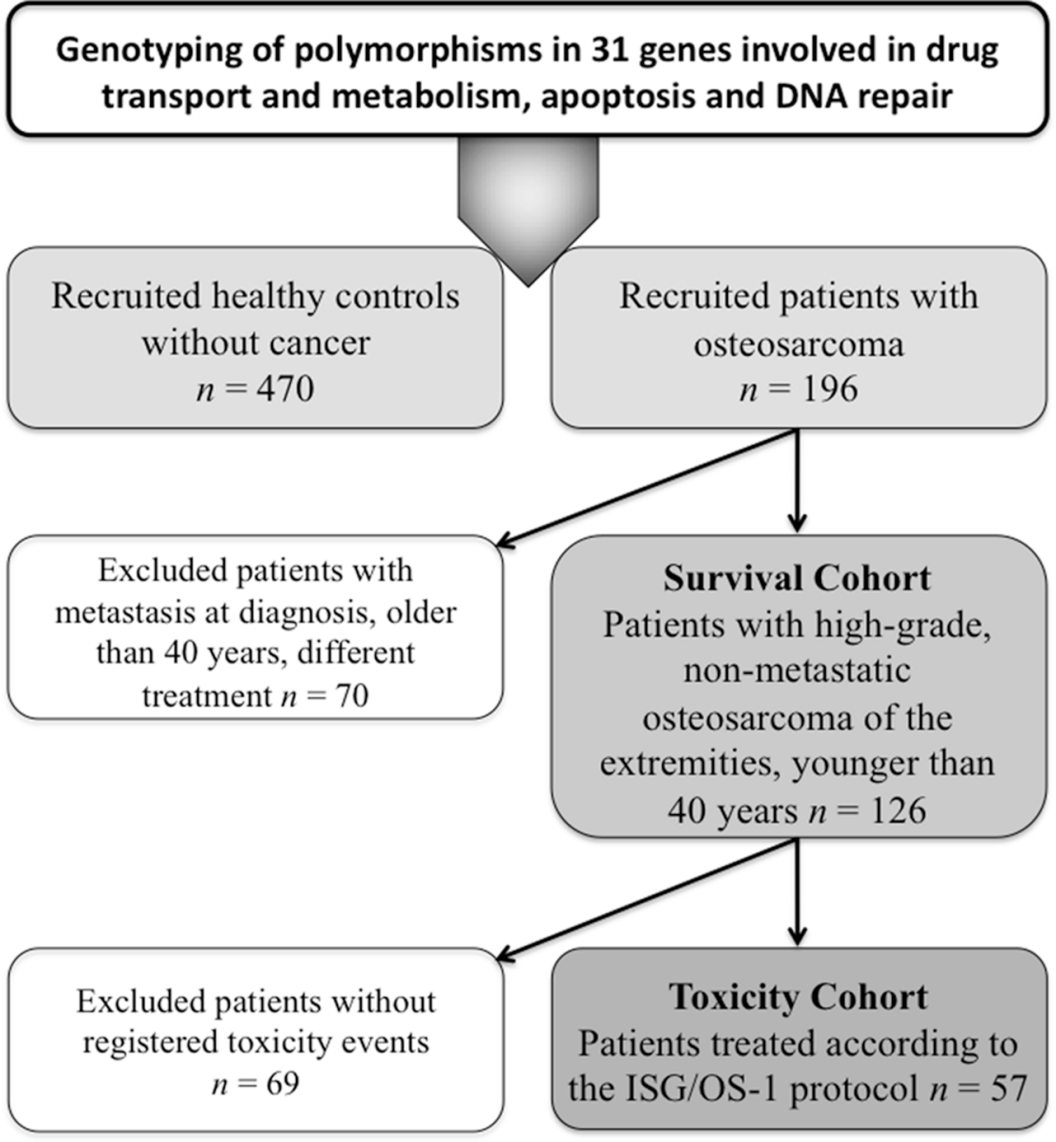
Flow diagram showing all cohorts included in genotyping and association analyses

A total of 196 patients with newly diagnosed HGOS (all recruited and treated at the Rizzoli Orthopaedic Institute, Bologna, Italy between 1984 and 2005) (Table 4) and 470 healthy controls (individuals without cancer at the time of enrollment, who were recruited at the National Cancer Institute, Aviano, Italy) were considered in order to identify polymorphisms associated with the development of HGOS (Figure 4). In order to match as much as possible the healthy controls to the patients cohort, all individuals over 40 years were excluded from the original pool of 1,000 healthy individuals. After this step, the control group showed a male/female ratio (1.55) and a median age (33 years) that were as close as possible to those of the patient cohort (male/female ratio: 1.65; median age 16 years). All individuals were of Caucasian ethnicity and had signed an informed consent. The study was approved by the institutional ethical review boards.

The impact of polymorphisms on clinical outcome was evaluated in a subgroup of 126 patients with conventional HGOS (high-grade tumor of the extremities, non-metastatic at diagnosis, arisen in patients younger than 40 years) treated with standard neoadjuvant chemotherapy protocols based on doxorubicin, high-dose methotrexate, cisplatin and ifosfamide in association to surgery (“survival cohort”). Median follow-up of the survival cohort was 126 months (range 73–345 months).

From this cohort of 126 patients, toxicity data were available for 57 patients (“toxicity cohort”) treated according to the ISG/OS-1 protocol [27]. Median follow-up of the toxicity cohort was 118 months (range 73–142 months). Toxicity assessment was performed before each chemotherapy cycle and graded from 0 to 4 according to Common Terminology Criteria for Adverse Events (CTCAE) v4.0.

### DNA isolation and polymorphism screening

Genomic DNA was extracted from frozen lymphocytes obtained from peripheral blood samples or normal muscular tissue using the High Pure PCR Template Preparation Kit (Roche Diagnostics GmbH, Mannheim, Germany). DNA quality and concentration were checked by spectrophotometry using the NanoDrop ND-1000 (NanoDrop Technologies, Wilmington, DE, USA).

Based on literature search (*www.ncbi.nlm.nih.gov/pubmed*), 45 polymorphisms and two haplotypes of 31 genes involved in transport, metabolism, activation and detoxification of the four drugs used in standard HGOS chemotherapy (methotrexate, doxorubicin, cisplatin and ifosfamide), as well as in apoptosis or DNA repair were selected for genotyping (Table 1).

Pyrosequencing (Biotage, Uppsala, Sweden), TaqMan genotyping with pre-designed SNP assays (Applied Biosystem, Foster City, CA, USA), PCR or restriction fragment length polymorphism (RFLP) analysis were performed as described (Table 8). Primer sequences and genotyping reaction details are available on request.

**Table 8:** Polymorphisms and methods of analysis

Main Gene Function	Gene Name_Polymorphism	Reference SNP Number	Method of Analysis	Reference
Transport	ABCB1_3435T/C	rs1045642	PSQ	[28]
	ABCB1_1236T/C	rs1128503	PSQ	[28]
	ABCB1_2677G > T/A	rs2032582	PSQ	
	ABCC2_1249A/G	rs2273697	PSQ	[28]
	ABCC2_-24A/G	rs717620	PSQ	[28]
	ABCC2_3972A/G	rs3740066	taqman assay	
	ABCG2_34A/G	rs2231137	taqman assay	
	ABCG2_421A/C	rs2231142	taqman assay	
	RFC_80A/G	rs1051266	PSQ	
DNA Repair	APE1_2197T/G	rs1130409	taqman assay	
	ERCC1_8092T/G	rs3212986	taqman assay	[29]
	ERCC1_19007T/C	rs11615	taqman assay	[29]
	hMLH1_676A/G	rs1799977	PSQ	[28]
	hMSH2_IVS12-6T/C	rs2303428	PSQ	[28]
	hOGG1_1245C/G	rs1052133	taqman assay	[28]
	XPD_35931T/G	rs13181	PSQ	[29]
	XPD_23591A/G	rs1799793	PSQ	[29]
	XPG_3508G/C	rs17655	PSQ	[29]
	XRCC1_28152A/G	rs25487	taqman assay	[28]
	XRCC3_18067T/C	rs861539	PSQ	[28]
Folate cycle	DHFR_Ins/Del	rs70991108	PCR	
	FOLR1_181delC	rs3833748	RFLP/PSQ	
	GGH_452T/C	rs11545078	taqman assay	
	GGH_401T/C	rs3758149	taqman assay	
	GGH_16T/C	rs1800909	taqman assay	
	MTHFD1_1958T/C	rs2236225	taqman assay	
	MTHFR_677T/C	rs1801133	PSQ	
	MTHFR_1298A/C	rs1801131	PSQ	
	SHMT_1420T/C	rs2273029	taqman assay	
	TYMS_28bp_VNTR	rs34743033	PCR/RFLP	[30]
	TYMS_1494del6	rs16430	PCR/RFLP	[30]
Apoptosis	ATM_40C/G	rs1800054	taqman assay	
	ATM_61A/G	rs1801516	taqman assay	
	MDM2_309T/G	rs2279744	PSQ	[14]
	p21_98A/C	rs1801270	taqman assay	
	TP53_PIN3_IVS3 + 16 bp	rs17878362	PCR	
	TP53_IVS2+38G/C	rs1642785	taqman assay	
	TP53_ex4+119G/C	rs1042522	PSQ/taqman assay	[14]
Drug metabolism	GSTT1_gen_null	rs_GSTT1_gen_null	PCR	[28]
	GSTM1_gen_null	rs_GSTM1_gen_null	PCR	[28]
	GSTP1_313A/G	rs1695	PSQ	
	CYP2C19*2_681A/G	rs4244285	PSQ	
	CYP2B6*6(516T/G + 785A/G)	rs3745274 and rs2279343	RFLP	
	CYP2B6*7(*6 + 1459T/C)	rs3745274 and rs2279343 and rs3211371	RFLP	
	CYP2C9*2_430T/C	rs1799853	PSQ	
	CYP2C9*3_1075A/C	rs1057910	PSQ	
	CYP3A4*1B_-392A/G	rs2740574	PSQ	

PSQ: pyrosequencing, PCR: polymerase chain reaction, RFLP: restriction fragment length polymorphism.

### Statistical analysis

Descriptive frequencies are provided for all variables in the data sets of the three cohorts. Hardy-Weinberg equilibrium was assessed by standard methods for each polymorphism in both the healthy controls and total patients cohort. No deviations were found in the healthy controls, whereas few deviations were found in the total patients cohort (Table 1). However, these polymorphisms were not excluded from the study because these deviations may originate from their biological impact in HGOS.

Pairwise linkage disequilibrium (LD) analyses for polymorphisms affecting the same gene were carried out by using the freely available LDPlotter software in both healthy controls and total patients cohort. Strong LD (with D′ > 0.95 and r^2^ > 0.75) was found only for the polymorphisms of GGH in both healthy controls and total patient cohort.

The Shannon entropy of the distribution of each polymorphism was computed in both the cohorts, patients and controls. Missing values were assigned a special symbol, which was then added to the alphabet of symbols used to denote the specific instance of each polymorphism.

The cluster index [31, 32] was computed to identify genotype groups within the same pathway (transport, DNA repair, folate cycle, apoptosis or detoxification) that behaved similarly in HGOS patients compared to healthy controls. Detailed description of the Shannon entropy and cluster index analyses are provided in the Supplementary_Information File.

Two-tailed Fisher's exact or chi-square tests were used to correlate genotypes with clinical and biological parameters and to evaluate statistical associations between different variables. Two-sided *p* values < 0.05 were considered as significant.

Logistic regression analysis was performed to estimate the odds ratios (OR) and the corresponding 95% confidence interval (CI) of each possible risk factor for the occurrence of adverse events.

Event-free survival (EFS) was calculated from diagnosis until the date of the first adverse event (relapse of the tumor at any site or death of disease) or the last follow-up examination. Survival curves for each polymorphism were drawn by the Kaplan-Meier method and statistical significance was determined by the log-rank test. The genotype status of the polymorphisms that were significantly associated with adverse survival was defined as “risk genotype”.

Toxicity assessment was performed for each chemotherapy cycle and included incidence evaluation of grade 4 leukopenia and thrombocytopenia, red blood cell and platelet transfusion (as a consequence of the hematological toxicity), fever, nausea and vomiting, stomatitis, grade 4 transaminases (hepatotoxicity), nephro- and neurotoxicity.

Polymorphisms significantly associated with adverse events in univariate analyses were also analyzed by Cox proportional hazards or multiple logistic regression analyses. Since this was an exploratory study, no multiple testing correction was applied, considering *p* values and CIs more informative for the interpretation of our data.

All statistical analyses were carried out by using the StatView 5.0.1 software. Power calculations with real input data obtained from the survival and toxicity cohorts and a type I error probability of 0.05 were performed using the freely available Power and Sample size program [33].

## SUPPLEMENTARY MATERIALS AND FIGURES


