# Supplementary material for: Candidate germline polymorphisms of genes belonging to the pathways of four drugs used in osteosarcoma standard chemotherapy associated with risk, survival and toxicity in non-metastatic high-grade osteosarcoma
Source: Oncotarget. 2016 Aug 22;7(38):61970–87. doi: 10.18632/oncotarget.11486 (PMC5308704; doi:10.18632/oncotarget.11486)
Supplement: Supplementary file 1 [file oncotarget-07-61970-s001.pdf]

Candidate germline polymorphisms of genes belonging to the pathways of four drugs used in osteosarcoma standard chemotherapy associated with risk, survival and toxicity in non-metastatic high-grade osteosarcoma

Supplementary Materials

Supporting information on information-theoretical measures applied for the analysis of polymorphism distributions

In this document we provide details on the measures based on information theory that have been used in this work. Two analyses were made: the first one is based on the comparison of the Shannon entropy of the polymorphism distribution between the two cohorts of patients and controls (see Section 1); the second kind of analysis makes use of the so-called cluster index and is illustrated in Section 2.

Shannon entropy

The notion of *information entropy* has been introduced by Claude Shannon in 1948 [1]. This measure is usually referred to as *Shannon entropy*, or simply entropy when clear from the context. Besides its applications in communication systems and computer science, the Shannon entropy has been also applied to data analysis in complex systems [2, 3].

In the following, we succinctly introduce the concept of Shannon entropy. Let us consider a simple system of which we observe the state at a given time. The observation can be modeled as a random variable X which can assume values from a finite and discrete domain D. If the observation is $x \in D$, which has a probability $P(x)$, then the information content of the observation is measured as $-log_2 P(x)$.[1] An improbable observation conveys more information than one associated to high probability. We can characterize the entropy of a system by averaging over its possible outcomes:

$$H(X) = -\sum P(x) \log P(x) \quad (1)$$

where the sum is over $x \epsilon D$.

In the definition of $H(X)$ we assume $0 \log 0 = 0$.

Intuitively, $H(X)$ measures the degree of randomness of the data produced by the system, or the process. For example, let's consider the case of a pure random binary variable: we have $X = \{0,1\}$ and $P(0) = P(1) = 0.5$. Hence, $H(X) = -2(0.5 \log(0.5))=1$. In general, for n symbols appearing with equal probability, we have $H(X) = \log(n)$, which is the maximum value. Conversely, if $P(0) \approx 1$ (so $P(1) = 1-P(0) \approx 0$), the entropy is quite small, as it is very likely that X will assume value 0 (see Figure 1). Therefore, a high entropy characterizes systems that show strong tendency to disorder, whilst it is low for ordered systems.

1 The usual unity measure in information theory is the bit, so logarithms are in base.

2 Hereafter, we will omit the base in the mathematical notation.

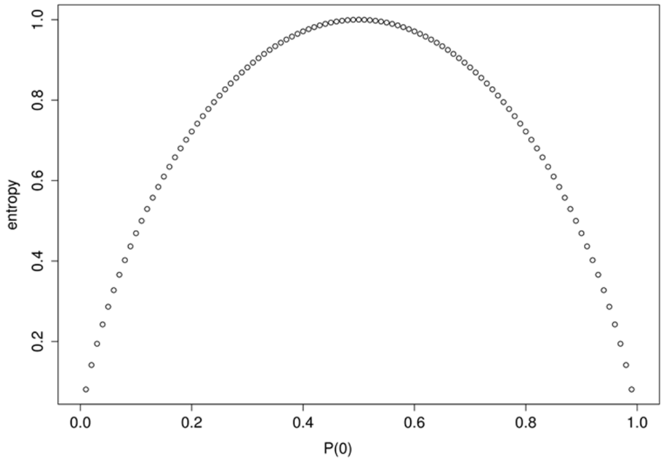

Supplementary Figure S1: Entropy values for a Bernoulli distributed stochastic variable.

Cluster index

The *functional cluster index* (cluster index, for short) has been introduced by Tononi et al. [5] as a mean to detect functional modules in the brain. Recently, it has also been successfully used to study complex dynamical systems and characterize their dynamical structure [6]. The intuition behind this index is that a high value should characterize a subset that is both strongly *integrated*, i.e. its components have strong similarity in the distribution of their values, and *segregated* with respect to the rest of the system, i.e. the distribution of the values assumed by variables in the subset is quite different w.r.t. the distribution of the variables outside the subset.

Let us consider a system modeled with a set U of N variables assuming finite and discrete values. The cluster index of a subset S of variables in U, $S \subset U$, as defined by Tononi [5], estimates the ratio between the amount of information integration among the variables in S and the amount of integration between S and U. These quantities are based on the Shannon entropy of both the single elements and sets of elements in U. As previously stated, the entropy of an element is defined as shown in equation (1). The entropy of a pair of elements X and Y is defined by means of their joint probabilities:

$$H(X,Y) = -\sum\sum P(x,y) \log P(x,y)$$

where the sums are over $x \in D_X$ and $y \in D_Y$

This last expression can be extended to sets of k elements considering the probability of occurrence of vectors of k values. In this work, we deal with observational data, therefore probabilities are estimated by means of relative frequencies.

The cluster index $C(S)$ of a set S of k elements is defined as the ratio between the integration $I(S)$ of S and the mutual information between S and the rest of the system $U{-}S$.

The integration of S is defined as:

$$I(S) = \sum H(x) - H(S)$$

$I(S)$ represents the deviation from statistical independence of the k elements in S. The mutual information $M(S;U{-}S)$ is defined as:

$$M(S;U{-}S) \equiv H(S) + H(S|U{-}S) = H(S) + H(U{-}S){-}H(S,U{-}S)$$

where $H(A|B)$ is the conditional entropy and $H(A,B)$ the joint entropy. Finally, the cluster index $C(S)$ is defined as:

$$C(S) = I(S)/M(S;U{-}S)$$

Since C is defined as a ratio, it is undefined in all those cases where $M(S;U{-}S)$ vanishes. In this case, the subset S is statistically independent from the rest of the system and it has to be analyzed separately. As $C(S)$ scales with the size of S, cluster index values of systems of different size need to be normalized. To this aim, a reference system is defined—the homogeneous system U_h—randomly generated according to the probability of each single state measured in the original system U. Then, for each subsystem size of U_h the average integration I_h and the average mutual information M_h are computed. Finally, the cluster index value of S is normalized by means of the appropriate normalization constant:

$$C'(S) = (I(S)/\langle I_h \rangle)/(M(S;U{-}S)/\langle M_h \rangle)$$

Furthermore, to assess the significance of the differences observed in the cluster index values, a statistical index is computed:

$$Tc(S) = (C'(S) - \langle C_h' \rangle)/\sigma(C_h')$$

where $\langle C_h' \rangle$ and $\sigma(C_h')$ are respectively the average and the standard deviation of the population of normalized cluster indexes with the same size of S from the homogeneous system. Cluster index analysis of polymorphisms

Cluster index analysis of polymorphisms

With the aim of finding clusters of polymorphisms differing between patients and controls cases, we computed the cluster index and its corresponding Tc for random samples of subsets of any size.[2] The result of this procedure is a list of candidate subsets of polymorphisms, ranked by Tc. If a subset is characterized by a high cluster index value w.r.t. a random distribution, then it is ranked in the first positions with a high Tc value.

REFERENCES

1. Shannon CE. A mathematical theory of communication. Bell Sys Tech J. 1948; 27:379–423.
2. Prokopenko M, Boschetti F, Ryan AJ. An information–theoretic primer on complexity, self-organization, and emergence. Complexity. 2008; 15:11–28.
3. Shalizi CR. Methods and techniques of complex systems science: An overview. arXiv:nlin/0307015, March 2006.
4. Frigge M, Hoaglin DC, Iglewicz B. Some implementations of the boxplot. The American Stat. 1989; 43:50–54.
5. Tononi G, McIntosh AR, Russel DP, Edelman GM. Functional clustering: Identifying strongly interactive brain regions in neuroimaging data. Neuroimage. 1998; 7:133–149.
6. Villani M, Roli A, Filisetti A, Fiorucci M, Poli I, Serra R. The search for candidate relevant subsets of variables in complex systems. Artificial Life. 2015; 21.

2 Given the combinatorial explosion of all the possible subsets, complete enumeration of all the possible subsets was not feasible. Therefore, 1000 random samples for each possible size were considered.
